# ﻿Genomic and ecological divergence support recognition of a new species of endangered *Satyrium* butterfly (Lepidoptera, Lycaenidae)

**DOI:** 10.3897/zookeys.1234.143893

**Published:** 2025-04-17

**Authors:** Zachary G. MacDonald, Julian R. Dupuis, James R. N. Glasier, Robert Sissons, Axel Moehrenschlager, H. Bradley Shaffer, Felix A. H. Sperling

**Affiliations:** 1 UCLA La Kretz Center for California Conservation Science, Institute of the Environment and Sustainability, University of California, Los Angeles, California, USA; 2 Department of Ecology and Evolutionary Biology, University of California Los Angeles, Los Angeles, California, USA; 3 Department of Biological Sciences, University of Alberta, Edmonton, Alberta, Canada; 4 Department of Entomology, University of Kentucky, Lexington, Kentucky, USA; 5 Wilder Institute/Calgary Zoo, Calgary, Alberta, Canada; 6 Resource Conservation, Waterton Lakes National Park, Waterton Park, Alberta, Canada; 7 IUCN SSC Conservation Translocation Specialist Group, Calgary, Alberta, Canada; 8 Panthera, New York, New York, USA

**Keywords:** Butterfly, Curiously Isolated Hairstreak, endangered species, genomics, Half-moon Hairstreak, niche divergence, Sagebrush Sooty Hairstreak

## Abstract

We describe a highly isolated population of hairstreak butterfly from Waterton Lakes National Park, Alberta, Canada, as a new species, *Satyriumcuriosolus***sp. nov.**, previously recognized as *Satyriumsemiluna* (Half-moon Hairstreak). We propose “Curiously Isolated Hairstreak” as the common name due to its disjunct and unusual distribution. Previous whole-genome analyses revealed *S.curiosolus* has extremely low genomic diversity and is highly divergent from the nearest *S.semiluna* populations in British Columbia and Montana, more than 400 km distant. Further analysis suggested prolonged inbreeding and isolation for up to ~40,000 years BP. Ecological niche modeling indicated that *S.curiosolus* occupies environmental conditions that are distinct from *S.semiluna*, suggesting niche divergence driven by long-term geographical and ecological separation. While host plant and ant associations have not been definitively resolved, they likely differ between *S.curiosolus* and *S.semiluna*. As part of this description, we provide whole-genome consensus sequences for each individual of the type series and identify 21,985 single nucleotide polymorphisms (SNPs) that are divergently fixed between *S.curiosolus* and *S.semiluna*, including 117 unlinked SNPs distributed across the genome as putative diagnostic markers. Previously listed as Endangered in Canada as the Waterton population of *S.semiluna*, *S.curiosolus* should retain this conservation status due to its extreme isolation, small population size, and flatlined genomic diversity. We propose species recognition as a testable hypothesis under the General Lineage Concept and recommend further research to explore the taxonomy, ecological relationships, and conservation of the greater species complex, including *S.curiosolus*, *S.semiluna*, and *S.fuliginosa*.

## ﻿Introduction

The northernmost populations of a North American butterfly, the Half-moon Hairstreak (*Satyriumsemiluna* Klots; sometimes called “Sagebrush Sooty Hairstreak”), have received recent study by MacDonald et al. (2025), but their taxonomic status remains in question. Although *S.semiluna* is “apparently secure” across its range in the USA (COSEWIC 2006, 2022; ECCC 2016; NatureServe 2024), the species’ northern range limit extends into Canada, where it is listed as Endangered under the “Species at Risk Act”. All but one Canadian population occur in south-central British Columbia, with an estimated aggregate abundance of 5,000–15,000 individuals. Based on continuity of both suitable habitat and the species’ occurrence records, British Columbia populations are presumably well connected to others south of the USA–Canada border and likely represent an example of political boundaries dictating protection rather than range-wide conservation concern. British Columbia populations have been recommended for downlisting to Threatened (COSEWIC 2022). The single other Canadian population persists on a ~300 ha alluvial fan (Blakiston Fan) in Waterton Lakes National Park, Alberta, where it is isolated from all other *S.semiluna* populations by a distributional gap of more than 400 km. This population was recently recommended for uplisting to Critically Endangered based on its uniqueness, small size, and considerable isolation (COSEWIC 2022).

The Alberta population is small, with genomically based estimates of contemporary effective population size (*N*_e_) around 500 individuals and surveys suggesting that between 1,000 and 10,000 adults fly annually (COSWEIC 2022; MacDonald et al. 2025). Aside from enigmatic island insect populations, such as the Lord Howe Island stick insect (*Dryococelusaustralis* (Montrouzier)) (Priddel et al. 2003) and some Hawaiian drosophilids (O’Grady and DeSalle 2018), few if any other insects have been documented with such a small population size and high degree of long-term isolation. The Alberta population’s environmental and ecological associations are also unique, adding to its scientific interest. Blakiston Fan receives an average summer precipitation of ~200 mm, while the locations of all other *S.semiluna* populations in the central-northern portion of the species' range receive between 32 and 154 mm (mean = 71 mm) (MacDonald et al. 2025). This difference in precipitation manifests in different habitat characteristics—Populations throughout British Columbia and the USA inhabit steppe-like habitats dominated by big sagebrush (*Artemisiatridentata* Nutt.). In contrast, occupied habitat at Blakiston Fan is best described as prairie/grassland dominated by sedges, grasses, and herbaceous plant species.

Another possible axis of niche divergence is larval host-plant association. Populations throughout British Columbia and the USA Pacific Northwest feed on silky lupine (*Lupinussericeus* Pursh) and possibly Pacific lupine (*Lupinuslepidus* Lindl.) (James and Nunnallee 2011), while the Alberta population feeds only on silvery lupine (*Lupinusargenteus* Pursh), even though *L.sericeus* is common at the site. Host associations of most other populations east of the continental divide in the USA are unknown and require investigation. Myrmecophily presents a third possible axis of niche divergence (MacDonald et al. 2025). Larvae of the Alberta population exhibit a mutualistic relationship with *Lasiusponderosae* Schär, Talavera, Rana, Espadaler, Cover, Shattuck & Vila. In British Columbia, *L.ponderosae* is absent in *S.semiluna* habitat, and larvae associate with *Formica* and *Camponotus* species. Similar associations with Formica and Camponotus have been observed in California (Runquist 2012).

Given the Alberta population’s small size and considerable isolation, inbreeding depression and loss of adaptive potential were identified by Parks Canada and the Half-moon Hairstreak Conservation Committee as likely threats to its long-term persistence. In these situations, genetic rescue is often assumed to be an effective conservation strategy (Storfer 1999; Weeks et al. 2011; Frankham et al. 2017; Ralls et al. 2020; Clarke et al. 2024). To assess whether genetic rescue is indeed appropriate for the Alberta population, MacDonald et al. (2025) generated the first chromosome-level genome assembly for the species and whole-genome resequencing data for the Alberta population, British Columbia populations, and the nearest USA population in Montana. Based on genetic divergence, environmental and ecological differences and a very long inferred history of isolation with no evidence of contemporary or recent gene flow, we, together with Parks Canada and the Half-moon Hairstreak Conservation Committee, determined that the Alberta population satisfies requirements of a distinct species that has long been on an independent evolutionary trajectory. Species-level recognition highlights the unique ecology and evolution of this butterfly, demonstrates a clear need for continued consideration under the “Species at Risk Act” and International Union for the Conservation of Nature, and provides an important case study on the utility of genomics in taxonomy. Genomics has an increasingly important role in taxonomic descriptions (Fennessy et al. 2016; Nater et al. 2017; Zhou et al. 2018; Stanton et al. 2019). However, chromosome-level genome assemblies for new species, along with whole-genome consensus sequences for the type series, remain rare (see Brandão‐Dias et al. 2022).

## ﻿Methods

All types (Fig. 2) are deposited in the University of Alberta E.H. Strickland Entomological Museum (**UASM**).

Here we summarize the taxonomically relevant methods of MacDonald et al. (2025). Eight individuals were collected from Blakiston Fan, Alberta, four from Richter Pass, British Columbia, three from Anarchist Mountain, British Columbia, and four near Red Lodge, Montana (Parks Canada Agency Research and Collection Permit WL-2021-39,020, Nature Conservancy Canada Research Permit NCC_BC_2021_SS001, and Nature Trust of British Columbia Permit #3461). Four Alberta individuals were used to generate a chromosome-level reference genome assembly using PacBio HiFi long-read sequencing (Pacific BioSciences, Menlo Park, California, USA) and Omni-C proximity ligation (Dovetail Genomics, Scotts Valley, California, USA).

Whole-genome resequencing of individuals from Blakiston Fan (*n* = 4) and the geographically nearest populations from Richter Pass (*n* = 4), Anarchist Mountain (*n* = 3), and near Red Lodge (*n* = 4) was performed on an Illumina NovaSeq S1 platform, with a target coverage of ~20×. Reads were aligned to our reference genome assembly and used to identify millions of single nucleotide polymorphisms (SNPs). Population structure and degree of admixture was assessed using PCA and the program “structure” (Pritchard et al. 2000) and genetic divergence among inferred genomic clusters was estimated using *F*_ST_ (Weir and Cockerham 1984). Genetic diversity was estimated using individual-based heterozygosity and nucleotide diversity (π), while the proportion of an individual’s genome within runs of homozygosity over 0.1 Mb (*F*_ROH_) served as an index of inbreeding. Historical effective population size (*N*_e_) was inferred from each individual's genome sequence using the pairwise sequentially Markovian coalescent (PSMC) (Li and Durbin 2011).

Nuclear whole-genome consensus sequences (fastq format) were generated for each individual using individual-level BAM files (produced in genotype calling) and the mpileup command (-C 50, -Q 30, and -q 30) in samtools (Danecek et al. 2021). This was piped into the vcf2fq command from vcfutils.pl using our genome assembly as the reference. Filtering included sites with inferred consensus quality < 20 and a read depth less than 8× or greater than two times each individual sample’s mean coverage, calculated from BAM files using samtools “depth”.

A series of MaxEnt models (Phillips et al. 2006) were generated to assess environmental and ecological associations of *S.semiluna* across the central and northern extent of the species’ range. To assess niche divergence of the Alberta population relative to others within this modelling extent, a MaxEnt model was trained excluding Alberta occurrences and used to predict habitat suitability for all *S.semiluna* occurrences, including Alberta. If the predicted suitability of Blakiston Fan was substantially lower than the locations of all other occurrences, niche divergence was inferred (Campbell et al. 2022).

## ﻿Results

**Figure 1.**
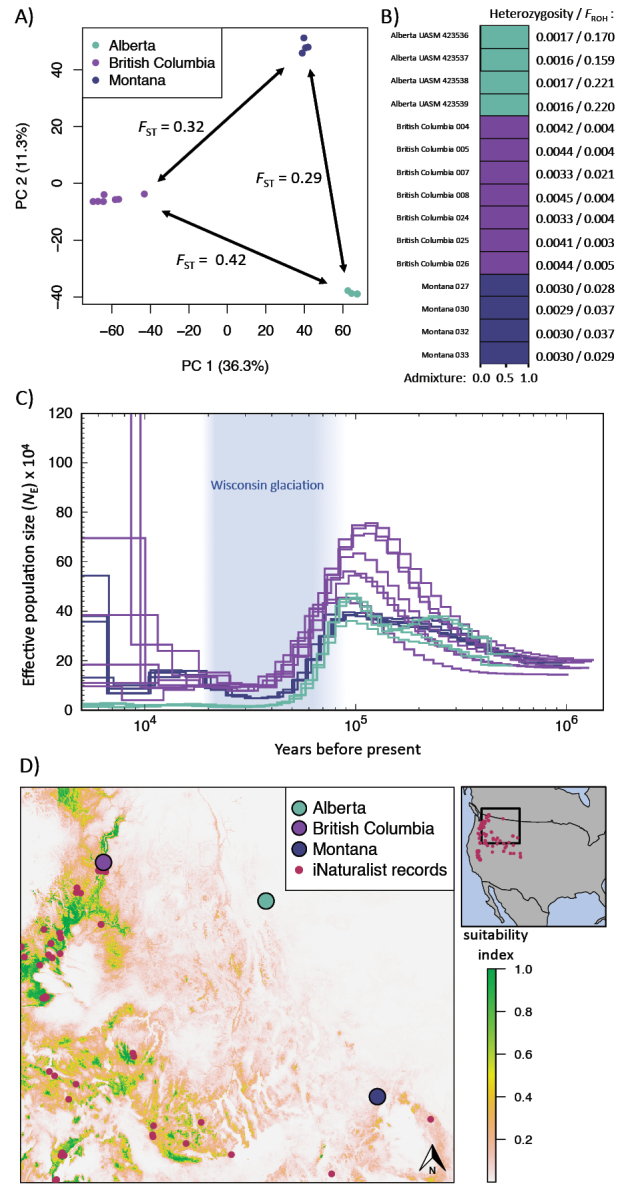
Summary of genomic and niche analyses from MacDonald et al. (2025)**A** principal component analysis (PCA) using a dataset of 108,283 physically unlinked single nucleotide polymorphisms (SNPs) separated sequenced individuals into three discrete clusters. Weir and Cockerham’s (1984)*F*_ST_ values are shown between the three clusters **B** clustering analyses using the program *structure* (Pritchard et al. 2000) of all individuals found an optimal *K* value of 2, splitting Alberta and Montana from British Columbia; hierarchical runs excluding British Columbia identified an optimal *K* value of 2 with virtually no admixture between Alberta and Montana. Here, we combine hierarchical runs into a single admixture plot. Average heterozygosity and an estimate of inbreeding (*F*_ROH_) for each individual is shown to the left of the admixture plot. Analyses of runs of homozygosity (ROH) and the proportion of each individual’s genome contained in ROH > 0.1 MB (*F*_ROH_) suggested that historical inbreeding has been much more prevalent in the Alberta population (mean *F*_ROH_ = 0.192) than in British Columbia (*F*_ROH_ = 0.006) or Montana (*F*_ROH_ = 0.033) populations, suggesting a long history of isolation **C** the pairwise sequentially Markovian coalescent (PSMC) from 2.5 mya until 5 kya of the three identified clusters, with each individual’s genome serving as an independent sample. Years before present is shown on the x-axis and estimated effective population size (*N*_e_) on the y-axis. The Alberta population flatlined between 1,000 and 5,000 individuals from 40 to 5 kya, indicating complete isolation. British Columbia and Montana both experienced substantial increases in *N_e_*, suggesting broad-scale connectivity. The approximate duration of the Wisconsin glaciation is shown in blue (Clayton and Moran 1982; Bischoff and Cummins 2001) D) Predicted *S.semiluna* habitat suitability, predicted using 17 environmental variables, landcover data, and various terrain indices. “Research-grade” iNaturalist occurrences and the collection locations of sequenced individuals, excluding the Alberta population, were used to parameterize the model. Blakiston Fan had suitability value of 0.003, while other *S.semiluna* populations inhabited areas of much higher suitability. Environmental conditions at Blakiston Fan are therefore atypical for the species, indicating niche divergence.

Our reference genome assembly was highly contiguous, spanning 1.25 Gb across 86 scaffolds, with an N50 of 56.2 Mb. Whole-genome resequencing of 15 individuals produced > 1.4 billion high-quality reads, yielding a dataset of 41,083,914 variants, with 23,889,641 SNPs retained after filtering. PCA and “structure” cleanly split all individuals into three populations with no evidence of admixture (Fig. 1a, b). *F*_ST_ values indicated substantial genetic divergence between Alberta and British Columbia (0.424), Alberta and Montana (0.292), and British Columbia and Montana (0.322). The two British Columbia sites showed no divergence, suggesting a high degree of gene flow (*F*_ST_ = −0.004). Mean heterozygosity was lowest in Alberta (0.083), compared to British Columbia (0.216) and Montana (0.154), and nucleotide diversity (π) was also lower in Alberta (0.003) than in British Columbia (0.008) and Montana (0.005) (Fig. 1b). This suggests much larger population sizes and broad-scale population connectivity in British Columbia and Montana compared to Alberta. Runs of homozygosity were 5–70 times more abundant in Alberta individuals, with individual *F*_ROH_ values averaging 0.192 in Alberta, 0.006 in British Columbia, and 0.033 in Montana (Fig. 1b). PSMC indicated that the Alberta population has been very small, isolated, and stable from 40 to 5 kya, with effective population size (*N*_e_) estimated between 1,000 and 5,000 individuals (Fig. 1c). In contrast, British Columbia and Montana populations experienced large expansions toward the end of this time period, suggesting broad-scale connectivity. MaxEnt models predicted habitat suitability with high accuracy (AUC_ROC_ = 0.94) and identified mean summer precipitation as the most important environmental variable predicting *S.semiluna* occurrences (Fig. 1d). When Alberta occurrences were excluded from model training, predictive accuracy increased (AUC_ROC_ = 0.97). Using this model to predict habitat suitability at Blakiston Fan resulted in an estimate of 0.003, much lower than the locations of all other *S.semiluna* occurrences. This was interpreted as evidence of niche divergence.

**Figure 2.**
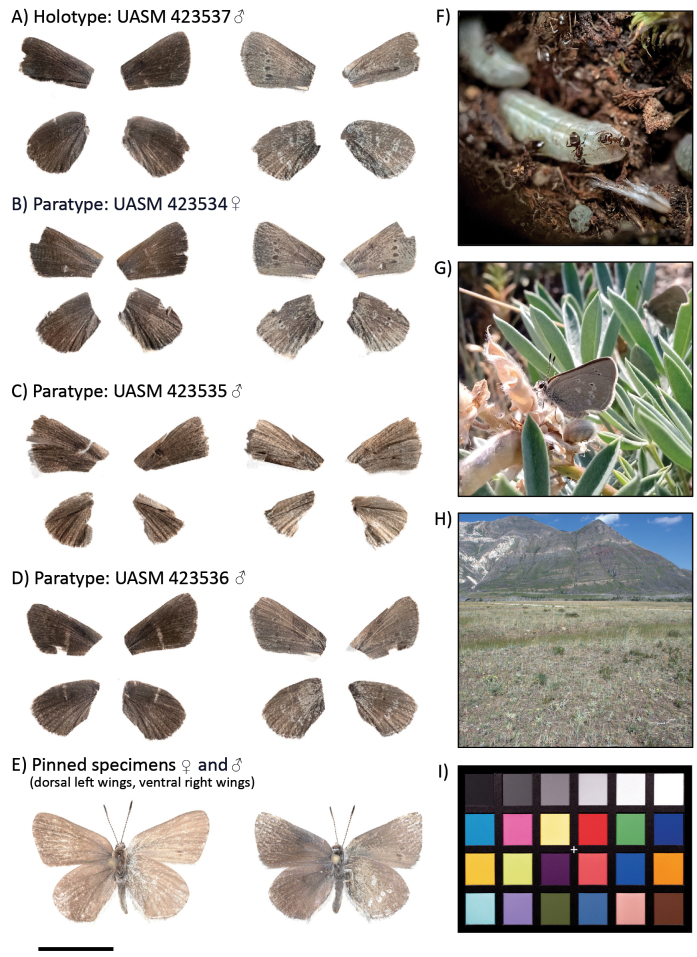
**A–D** dorsal and ventral wing surfaces of the *Satyriumcuriosolus* type series. Bodies of these specimens were used in genomic DNA extractions. Sequence data were used to generate a whole-genome consensus sequence for each specimen. Metadata for each specimen are given under “Type material” **E** composite photographs of pinned specimens (female left, male right), showing the dorsal wing surface on the left forewing and hindwing and the ventral wing surface on the right forewing and hindwing **F***S.curiosolus* larvae being attended to by *Lasiusponderosae* ants **G** a freshly eclosed *S.curiosolus* on silvery lupine (*Lupinusargenteus*); H) Photograph of Blakiston Fan, Alberta, Canada **I** Calibrite ColorChecker Classic, photographed with the same setup and settings used to photograph the type series. The scale bar (bottom left) is 1 cm, against which **A–E** are scaled.

Genetic divergence, environmental and ecological divergence, and a very long history of isolation with no evidence of contemporary or recent gene flow are sufficient to recognize the Alberta population as a distinct taxonomic entity. We propose its recognition as a new species.

### ﻿ Satyrium curiosolus

Taxon classificationAnimaliaLepidopteraLycaenidae

MacDonald, Dupuis, Glasier, Sissons, Moehrenschlager, Shaffer & Sperling sp. nov.

C9102B72-7001-527A-AF66-6D625BC39730

https://zoobank.org/92A9AB4C-2C9B-47CD-AB08-F248D92A078D

#### Type locality.

Canada: Alberta, Waterton Lakes National Park, Blakiston Fan, 49.068, −113.877.

#### Type material examined.

***Holotype*.** 1 1 [white label] “CANADA: Alberta, Waterton Lakes National Park, Blakiston Fan (Marquis), 49.068, −113.877 (WGS84), 14-Jul-2021, J. Glasier; 14008, Saytrium_curiosolus_016”; [white label] “UASM 423537”; [red label] “Holotype *Satyriumcuriosolus*”. BioSample: SAMN45172752.

***Paratypes*.** 1 1[white label] “CANADA: Alberta, Waterton Lakes National Park, Blakiston Fan (Hay Barn), 49.079, −113.866 (WGS84), 14-Jul-2021, J. Glasier; 14002, Saytrium_curiosolus_009”; [white label] “UASM 423534”. BioSample: SAMN45172749 • 1 1[white label] “CANADA: Alberta, Waterton Lakes National Park, Blakiston Fan (Hay Barn), 49.078, -113.869 (WGS84), 14-Jul-2021, J. Glasier; 14003, Saytrium_curiosolus_010”; [white label] “UASM 423535”. BioSample: SAMN45172750 • 1 1[white label] “CANADA: Alberta, Waterton Lakes National Park, Blakiston Fan (Hay Barn), 49.076, −113.869 (WGS84), 14-Jul-2021, J. Glasier; 14006, Saytrium_curiosolus_012”; [white label] “UASM 423536”. BioSample: SAMN45172751.

#### Description.

The morphological description follows Mattoon and Austin’s (1998: 685) description of *Satyriumfuliginosumsemiluna* Klots, which is now recognized as *Satyriumsemilunasemiluna* Klots (Warren 2005).

A small, drab butterfly. As with many *Satyrium*, wings predominately brownish or dark brown dorsally (fading to light brown with age), lacking any hint of blue, and lacking tails. Males with strong dorsal scent pad of androconial scales, shared with *S.semiluna*, but lacking in *S.fuliginosa* (W.H. Edwards) (Warren 2005). Ventral wing surface light brown with grey overscaling along margins, and large black postmedial spots slightly outlined in white (reduced in hindwings). Females slightly larger and paler ventrally.

#### Diagnosis.

Males with small wingspan (<25 mm vs > 30 mm) and less conspicuous ventral spotting than *S.semiluna* (Kondla 2004). Due to the cryptic nature of the species, identification without reference to source locality is most reliably achieved by DNA as follows, with representative (see Remarks section) diagnostic single nucleotide polymorphisms (SNPs) that are fixed for *S.curiosolus* (formatted as scaffold: position[*S.curiosolus* allele/*S.semiluna* allele]: ScvBUXZ_1.HRSCAF10: 1568673[C/A], 12071375[C/A], 22597556[G/C], 33128087[T/G], 43633436[G/C], 54341325[T/A]; ScvBUXZ_11.HRSCAF312: 939246[C/T], 11508752[A/T], 23223489[T/C], 34114361[T/C]; ScvBUXZ_12.HRSCAF324: 2725346[G/T], 13277889[C/A], 23561701[T/A], 34983585[G/A]; ScvBUXZ_15.HRSCAF369: 604178[A/G], 10688359[A/G], 22742875[A/G], 33306760[T/C], 43372888[A/C]; ScvBUXZ_16.HRSCAF394: 1798098[A/G], 13584119[G/C], 24134185[G/A], 36106687[A/G]; ScvBUXZ_18.HRSCAF419: 1271585[C/T], 12498609[T/A], 22674145[A/C], 32848269[A/G], 43126131[A/G], 53221485[G/A], 63907163[T/A]; ScvBUXZ_20.HRSCAF485: 1278399[C/A], 11291064[A/G], 22201816[G/T], 34957432[C/T], 45290319[A/G], 56419490[G/A], 70868124[T/C], 82626599[G/C]; ScvBUXZ_21.HRSCAF503: 3759737[G/A], 14004891[T/C], 24159643[A/T], 34796558[A/G], 46064578[A/C]; ScvBUXZ_22.HRSCAF557: 1241910[T/C], 11429001[A/G], 22696299[A/G], 32721285[T/C], 43059425[T/G], 53074410[A/G], 63089033[T/G], 74842831[G/T]; ScvBUXZ_23.HRSCAF563: 1626681[A/G], 12005836[C/A], 22106743[T/G], 32206731[A/C], 42322988[A/C]; ScvBUXZ_27.HRSCAF638: 617786[A/G], 10908292[T/G], 20909876[T/C], 31703436[A/C], 42607435[A/G], 52716659[T/C], 63015994[A/G]; ScvBUXZ_3.HRSCAF45: 40925[T/C], 10620232[A/C], 20622161[A/C]; ScvBUXZ_33.HRSCAF736: 1025752[C/T], 11845689[T/C], 21926431[A/G], 33073314[T/C], 43525185[C/A]; ScvBUXZ_36.HRSCAF762: 496718[G/T], 12599380[T/C], 22623129[A/C], 32789532[T/G], 43886779[C/T], 53941324[T/C]; ScvBUXZ_37.HRSCAF777: 1137238[A/C], 11176395[C/T], 21849140[A/G], 36719032[G/T], 46746341[A/C], 57389780[G/A]; ScvBUXZ_4.HRSCAF59: 2539048[A/C], 15455375[T/G], 25460453[T/C], 35798787[C/A], 46323982[A/C], 59292711[A/G]; ScvBUXZ_41.HRSCAF810: 342264[A/G], 10823670[C/T], 21337909[T/C], 31863338[G/C], 41915594[A/T], 52015696[T/G]; ScvBUXZ_48.HRSCAF855: 2065895[T/C], 12340828[G/C], 22489657[A/G], 33211401[A/G], 43237667[T/A]; ScvBUXZ_5.HRSCAF87: 653322[A/G], 11521727[C/G], 22249471[A/G], 32408138[T/A], 42462727[T/G], 52471692[A/G]; ScvBUXZ_54.HRSCAF883: 1953965[C/A], 12005082[A/G], 22185366[C/T], 32223581[C/T]; ScvBUXZ_6.HRSCAF109: 2728984[T/C], 14807116[G/A], 25931083[T/C]; ScvBUXZ_9.HRSCAF216: 1632867[C/G], 14935276[A/G], 26523755[C/G], 37425842[T/A]

#### Genomic sequence of the holotype.

BioSample: SAMN45172752; whole-genome consensus sequence available on Dryad: https://doi.org/10.5061/dryad.sf7m0cgj2.

#### Distribution.

Currently known only from Blakiston Fan, Alberta, Canada, approximately 300 ha in area.

#### Seasonality.

Eggs overwinter before hatching in early spring in late April or early May. Larvae can first be found in early May, develop through four instars, pupate in July (at the base of *L.argenteus*, often under the previous year’s stems in ant galleries), and then emerge after about two weeks of pupation in July to mid-August.

#### Ecology.

Restricted to Blakiston Fan, a 300-ha area of course-textured alluvial fan at an elevation of ~1,300 m. The habitat of *S.curiosolus* is short-grass prairie with abundant *L.argenteus*, *L.sericeus*, and yellow buckwheat (*Eriogonumflavum* Nutt.?). This habitat differs from that of *S.semiluna*, in that is it lacking big sagebrush (*A.tridentata*). *Satyriumsemiluna* is associated with *A.tridentata* to the point that, in the USA, the butterfly’s common name is the Sagebrush Sooty Hairstreak. Another notable difference is that *S.semiluna* populations generally inhabit hillsides or mountainsides, while *S.curiosolus* inhabits an alluvial fan in the middle of a montane valley.

Unlike previous reports stating that that *S.curiosolus* uses both local lupine species as plant hosts (COSEWIC 2006, 2022; ECCC 2016), our surveys found that they only use *L.argenteus*. Out of ~500 larvae detected in repeated surveys throughout 2020–2024, all were on *L.argenteus*. *Satyriumsemiluna* populations on the west side of the Rocky Mountains, and presumably those throughout the central USA, feed on *L.sericeus*. These populations may also feed on *L.lepidus* (James and Nunnallee 2011), but in extensive surveys throughout British Columbia in 2021–2024, we have not observed any such association (Glasier pers. obs.). *Satyriumcuriosolus* larvae feed on new buds and stems at the base of lupines and commonly hide under the woody stems from the previous year when not feeding.

At Blakiston Fan, all *S.curiosolus* larvae observed in 2021–2024 surveys were closely associated with *Lasiusponderosae* ant colonies (identified using Glasier et al. 2013 and Schär et al. 2022). Ants groomed and protected the larvae, and larvae were observed to retreat into ant colonies when threatened. Other ant species seen interacting with larvae at Blakiston Fan include *Formicaargentea* and *Formicalasioides* (ants identified using Glasier et al. 2013. However, these interactions appeared to be more opportunistic, as these larvae observed were still primarily associated with a *L.ponderosae* colony. We have also observed *S.curiosolus* larvae pupating in the galleries of *L.ponderosae* colonies at the base of *Lupinusargenteus* plants. During our butterfly surveys in 2021–2024 in British Columbia, no *Lasius* species were found attending *S.semiluna* larvae. Instead, *Camponotusvicinus*, *Formicaobscuripes*, and *Formicaargentea* were observed interacting with larvae, and several pupae were found in a *Camponotusvicinus* nest at the base of a *L.sericeus*. In California, *Camponotus* and *Formica* attendants were also reported (Runquist 2012).

*Satyriumcuriosolus* fluctuates in abundance from year to year, with genomically based estimates of contemporary effective population size (*N*_e_) around 500 (MacDonald et al. 2025) and surveys suggesting that between 1,000 and 10,000 adults fly annually (COSEWIC 2006, 2022; unpublished data). Based on our observations from 2020–2024, the *S.curiosolus* flight period occurs during July to mid-August and lasts about two weeks. Adults are most frequently observed as they perch, sunning themselves on buckwheats, lupines, and shrubby cinquefoil (*Dasiphorafruticosa*). Males tend to spend more time on alpine buckwheat, while females tend to spend more time on lupines. Mating occurs at any time of day and may last several hours. Females lay an unknown total number of eggs but have been observed laying eggs singly or in small clusters, in the soil around the base of *L.argenteus* and/or near the entrance of *L.ponderosae* nests.

#### Etymology.

The specific epithet *curiosolus* derives from the Latin “*curiosus*” meaning curious and “*solus*” meaning to be alone or isolated, and it is to be treated as a noun in apposition. We suggest the common name “Curiously Isolated Hairstreak”.

#### Remarks.

Using our reference genome assembly (NCBI JASDAZ000000000) and whole-genome resequencing data for 15 individuals, we identified 21,985 SNPs across 22 scaffolds that were fixed for alternate nucleotides between individuals from Alberta and those from Montana and British Columbia. The 117 SNPs included in this description result from thinning to one SNP per 10 Mb (using --thin option in vcftools v0.1.16, Danecek et al. 2011) to ensure that they are evenly spaced across the genome and likely physically unlinked. DNA barcodes (mitochondrial gene cytochrome oxidase subunit I) have been shown to be identical between populations of *S.semiluna* from Alberta, British Columbia, and Washington (COSEWIC 2006), and haplotype sharing (in cytochrome oxidase subunit II) has been observed more broadly between *S.semiluna* and *S.fuliginosa* (Runquist 2012); both observations suggest that mitochondrial/nuclear discordance exists within the genus. Taken together with other systematic studies with broader taxonomic sampling (Robbins et al. 2022), these data also provide support for th inclusion of *S.curiosolus* within *Satyrium*. Nuclear whole-genome consensus sequences for each individual of the type series are available at: https://doi.org/10.5061/dryad.sf7m0cgj2.

## ﻿Discussion

*Satyriumcuriosolus* warrants recognition as a distinct taxonomic entity. We evaluated whether to describe it as a subspecies or a species based on two main criteria. Braby et al. (2012) defined subspecies by the combination of partial isolation of allopatric lineages, phenotypic distinctiveness, and at least one fixed, diagnosable character. This definition is rooted in the General Lineage Concept (GLC), which considers species as independently evolving lineages supported by multiple lines of evidence including criteria often associated with various species concepts (De Queiroz 1998, 2007). To ensure an objective comparison, we assess explicit criteria from these concepts (Table 1), taking them at face value as described in their original publications. While one interpretation of the GLC is that it lacks any specific criteria, like intrinsic reproductive isolating mechanisms or fixed morphological characters (De Queiroz 1998), practical application necessitates that we identify and score multiple criteria in making a species/subspecies determination. Thus, we focus on criteria used by alternative species concepts relevant to the “grey zone” of speciation (De Queiroz 2007).

**Table 1.** Criteria and properties used to define subspecies following Braby et al. (2012) and species following concepts unified under the General Lineage Concept (De Queiroz 1998, 2007, and references therein for various properties of alternative species concepts). “This study” represents whether a criterion/property is satisfied in this system (“1”), not satisfied (“0”), or unknown with the data at hand (“?”).

Concept/definition	Criteria/property	This study
Subspecies, Braby et al. (2012)	Partially isolated lineages	0
	Allopatric	1
	Phenotypically distinct	?*
	≥1 fixed, diagnosable character state (assumed correlation to evolutionary independence)	1
Species, General Lineage Concept (De Queiroz 1998, 2007)		
	Reproductive incompatibility/character displacement	?**
	entirely allopatric	1
	Mate recognition systems	?
	Ecologically distinct	1
	Monophyly	1
	Lack of gene flow	1
	Morphologically diagnosable	?*
	Genetically diagnosable	1
	Ecologically diagnosable	1

*Morphological diagnosability is generally possible with many specimens in series, but confident morphological delimitation of single specimens of *S.curiosolus* from *S.semiluna* may be difficult without other data (collection locality, DNA data). **MacDonald et al. (2025) inferred that the Alberta *Satyrium* population in question (described here as *S.curiosolus*) would likely experience outbreeding depression if it mated with other populations, which may be interpreted as a form of reproductive isolation; experimental crosses are needed to futher assess this inference.

Allopatry and isolation are critical properties for many subspecies and species concepts. Here, all evidence indicates that divergence between *S.curiosolus* and the geographically nearest *S.semiluna* populations is non-clinal, with no evidence of contemporary or recent gene flow. *Satyriumcuriosolus* is completely isolated today, and coalescent-based analyses suggest this isolation may extend up to 40,000 years BP. Given this considerable isolation, traditional considerations of potential or actual reproductive isolation (Mayr 1942) are difficult to apply, and secondary contact with *S.semiluna* is improbable given both species’ relatively low vagility and the magnitude of range shift required (MacDonald et al. 2024). From a geographical standpoint, there is no possibility of hybridization and gene flow. This is a hypothesis that could be falsified by the discovery of genetic and environmental/ecological intermediates between *S.curiosolus* and *S.semiluna*. We consider this unlikely, given the prominent butterfly survey effort in the region. Nonetheless, we and others will continue to search for undiscovered populations. *Satyriumcuriosolus* exhibits ecological distinctiveness, including unique environmental, host plant, and ant associations. Morphological distinctiveness was not extensively investigated but has been suggested by other species experts—Kondla (2004) noted that males from Blakiston Fan (*S.curiosolus*) have a smaller wingspan than other *S.semiluna* populations investigated (<25 mm vs > 30 mm) and less conspicuous ventral spotting. Beyond size and wing pattern, our most concrete diagnosable characters include 21,985 SNPs that are divergently fixed for alternate nucleotides between *S.curiosolus* and the nearest *S.semiluna* populations. For simplicity, we identified a subset of 117 SNPs evenly spaced across the genome. Future resequencing of additional *S.curiosolus* and *S.semiluna* individuals may reveal some of these SNPs to be “near fixed” even though they are fixed in our sample set—this would not invalidate them as diagnostic characters, but simply suggest that they have not evolved to complete fixation. As with any set of diagnostic characters, our evaluation of genomic diagnostic characters may change as new information becomes available.

Complete absence of gene flow, and a long history of isolation combined with genomic, environmental, and ecological differentiation, satisfies many of the criteria associated with alternate species concepts and unified under the GLC, notably those originating from the phylogenetic species concept (including variants introduced by Rosen 1979; Baum and Shaw 1995; Nelson and Platnick 1981; Cracraft 1983), the ecological species concept (Van Valen 1976), and the evolutionary species concept (Simpson 1951; Wiley 1978). The alternative—retaining *S.curiosolus* as a subspecies—would imply ongoing or potential gene flow, which is demonstrably absent, making species recognition the most taxonomically defensible classification (Braby et al. 2012). Still, we extensively debated the appropriate classification, initially considering subspecies as a more prudent and conservative option, anticipating that further evidence and analyses may substantiate species-level recognition. However, assuming that subspecies rank is the more prudent or conservative classification was potentially problematic. This assumption treats subspecies as an intermediate stage rather than objectively evaluating evolutionary independence based on multiple lines of evidence. Subspecies status often reflects structured intraspecific variation with some degree of contemporary or recent gene flow, not clear lineage separation (Mayr 1963; Braby et al. 2012). Under our view of the GLC, species recognition is a testable hypothesis, not a permanent designation, and should be based on the strength of evidence rather than a default bias toward subspecies as a sort of evolutionary null. Treating subspecies as a holding category creates an asymmetrical burden of proof, requiring disproportionately strong evidence for species recognition while subspecies designations persist under weaker criteria. This bias could obscure evolutionary significance and delay the recognition of independent lineages by misrepresenting biodiversity.

### ﻿Future taxonomy and conservation

Further taxonomic and phylogenomic research on this species complex should incorporate *S.fuliginosa*, which is currently thought to be restricted to California and southern Oregon, where it is sympatric and may hybridize with *S.semiluna* (Runquist 2012). These putative sister species were historically distinguished by the presence of a male forewing scent patch in *S.semiluna* (a synapomorphy of *Satyrium*; see Martins et al. 2019), which is entirely absent in *S.fuliginosa* (Warren 2005), as well as the generally browner wing coloration of *S.fuliginosa* compared to the greyer *S.semiluna*. However, the validity of these distinct taxa has been questioned (Runquist 2012), and morphological outlier populations—greyer *S.fuliginosa* and browner *S.semiluna*—have been identified in western California (Warren 2005; Runquist 2012). Despite this complexity, the male scent patch of the Blakiston Fan population aligns it with the *semiluna* group *sensu* Matton and Austin (1998), rather than the *fuliginosa* group. This distinction, along with the fact that *S.fuliginosa* is more geographically distant from *S.curiosolus*, suggests that only comparisons between *S.curiosolus* and northern *S.semiluna* populations are pertinent to the taxonomic revision proposed here. However, to understand the full extent of differentiation and diversity between and within related groups of species within this genus, we recommend that future research include the full geographical extent of known populations of all three taxa as well as other *Satyrium* species to provide phylogenetic context (e.g. *S.calanus* Hübner, *S.californica* W.H. Edwards, and *S.sylvinus* Boisduval; Runquist 2012). We also recommend further sequencing to confirm the reported identical mitochondrial haplotypes observed between *S.curiosolus* and *S.semiluna* (COSEWIC 2006) and shared haplotypes between *S.semiluna* and *S.fuliginosa* (Runquist 2012). When such comparative data are available, the second component of the species concept of Sperling (2003) can be applied, which uses the extent of divergence between recognized sister species in parapatry or sympatry to calibrate the threshold for species recognition of allopatric populations.

We note that *S.fuliginosa* is frequently referred to as “*S.fuliginosum*” in species status assessments of *S.semiluna* (e.g. COSEWIC 2006, 2022, with inconsistent usage within single documents), survey reports (e.g. Kondla 2003), and the original *S.semiluna* description (Matton and Austin 1998). Originally described as *Lycaenafuliginosa* (W.H. Edwards), the species was later reclassified into *Satyrium* and the use of “*S.fuliginosum*” stems from the inference that *Satyrium* is a neuter genus name, requiring gender agreement of species epithets (Scudder 1876). However, global lepidopterists’ societies have maintained original orthography, even in cases of gender incongruence (Sommerer 2002; van Nieukerken et al. 2019). We have chosen to use the name *S.fuliginosa*, as reflected in Pelham’s (2023) catalogue of butterflies of the United States and Canada.

Based on genomic and ecological divergences, MacDonald et al. (2025) recommended that genetic rescue, involving the translocation of individuals from other *S.semiluna* populations to Blakiston Fan, is more likely to be harmful than helpful at present. Parks Canada has accepted this recommendation and is managing *S.curiosolus* in isolation. The taxonomic distinctiveness of *S.curiosolus* suggests substantial risk of outbreeding depression or reproductive incompatibility if genetic rescue involving *S.semiluna* were attempted. However, the low genetic diversity that characterizes *S.curiosolus* may hinder adaptation under accelerating climate change. In the future, the trade-off between a lack of adaptive capacity and outbreeding depression may shift in favor of managed introgression with *S.semiluna* populations, should they be found to be reproductively compatible. Genetic rescue can introduce beneficial genomic variation that is integral to rapid adaptation (Edelman and Mallet 2021) and hybrid vigor (“heterosis”) has long been recognized as a possible benefit of hybridization, even between distinct species (Darwin 1859; Birchler et al. 2003; Lippman and Zamir 2007; Parmesan et al. 2023). Hybridization is common between species, and should not always be viewed as undermining species-level recognition (Mallet 1995; Sperling 2003; Taylor et al. 2015). The possibility of genetic rescue—should be regularly revisited as local climatic and habitat conditions at Blakiston Fan continue to change.

## Supplementary Material

XML Treatment for Satyrium curiosolus

## ﻿Additional information

### Author ORCIDs

Zachary G. MacDonald https://orcid.org/0000-0002-7966-5712

Julian R. Dupuis https://orcid.org/0000-0002-6989-9179

James R. N. Glasier https://orcid.org/0000-0002-3449-9183

Axel Moehrenschlager https://orcid.org/0000-0003-2789-0376

H. Bradley Shaffer https://orcid.org/0000-0002-5795-9242

Felix A. H. Sperling https://orcid.org/0000-0001-5148-4226

### Data availability

All of the data that support the findings of this study are available in the main text.
